# Cured but not well — haematological cancer survivors’ experiences of chemotherapy-induced peripheral neuropathy in everyday life: a phenomenological-hermeneutic study

**DOI:** 10.1007/s11764-024-01612-4

**Published:** 2024-05-14

**Authors:** Mette Louise Roed, Marianne Tang Severinsen, Eva Futtrup Maksten, Lone Jørgensen, Helle Enggaard

**Affiliations:** 1 Department of Haematology, Clinical Cancer Research Center, Aalborg University Hospital, Aalborg, Denmark; 2 Department of Clinical Medicine, Aalborg University, Aalborg, Denmark; 3 Clinical Nursing Research Unit, Aalborg University Hospital, Aalborg, Denmark; 4 Clinic Cancer Research Unit, Aalborg University Hospital, Aalborg, Denmark

**Keywords:** Cancer, Chemotherapy-induced peripheral neuropathy, Survivorship, Everyday life, Qualitative research, Thematic analysis

## Abstract

**Purpose:**

To explore haematological cancer survivors’ experience of chemotherapy-induced peripheral neuropathy (CIPN) in everyday life.

**Methods:**

Data were generated by means of individual semi-structured interviews with 12 haematological cancer survivors who experience CIPN after completion of treatment. Data were analysed using thematic analysis.

**Results:**

The thematic analysis yielded an in-depth description of the experience of CIPN symptoms and the influence of the symptoms on everyday life as being unwell despite being cured. Four main themes emerged from the analysis: (1) *A diffuse and contradictory sensation which is impossible to ignore in everyday life*, (2) *Not feeling well, even though I’m cured*, (3) *Living with CIPN, despite limitations*, and (4) *An invisible companion, that everybody knows about.*

**Conclusion:**

The findings shows that survival from haematological cancer does not always equal well-being, as experiencing CIPN has extensive consequences on everyday life. CIPN affects haematological cancer survivors’ transition to an ordinary everyday life, with disturbances in the physical function, daily activities, social relationships, psychological aspects, and work ability. As a diffuse and contradictory symptom, CIPN appears as an invisible companion that leads to a feeling of being alone.

**Implications for Cancer Survivors:**

A better and deeper understanding of haematological cancer survivors’ experience of CIPN in everyday life may improve communication, guidance, and treatment of CIPN symptoms. The results suggest a need for interventions and strategies to accommodate the gap in practice and to address the impact of CIPN in everyday life.

**Supplementary Information:**

The online version contains supplementary material available at 10.1007/s11764-024-01612-4.

## Introduction

Survivorship among patients diagnosed with haematological cancer is of increasing importance due to improved treatment options and long-term survival [1–3]. Chemotherapy is an integral part of haematological cancer treatment, but for some survivors, it may affect the nervous system resulting in sensory, motor, and autonomic disturbances, overall described by the term chemotherapy-induced peripheral neuropathy (CIPN) [4, 5]. Although it is well known that CIPN can affect haematological cancer survivors, we lack knowledge of the influence it may have on the survivors’ everyday life.

CIPN has developed into a widespread late effect of chemotherapy, affecting more than one-third of all haematological cancer survivors even after end of treatment [6]. The symptoms may develop during treatment and may occur months or years after cessation of chemotherapy [4]. CIPN is a potentially debilitating late effect which arises in the upper and lower extremities and include symptoms like pain, tingling, numbness, burning, hypersensitivity to cold and heat, and problems with balance, walking, and tasks requiring motor skills [7, 8]. Current research shows that CIPN has a negative impact on quality of life among haematological cancer survivors because the symptoms cause disturbances in the physical function, daily activities, social relationships, psychological aspects, and work ability [9–13]. Despite this, its significance remains under-researched among haematological cancer survivors. In addition, no convincing evidence about how to prevent, reduce, or treat CIPN is available and CIPN remains a challenge for survivors as well as healthcare professionals [5, 8]. At present, only duloxetine is a recommended treatment for the painful CIPN [14, 15].

A meta-synthesis on cancer survivors’ experiences of CIPN after treatment completion shows that CIPN has an impact on many aspects of everyday life [16]. However, in this meta-synthesis, only two of the 13 studies included haematological cancer survivors [17, 18]. These two studies showed that cancer survivors with CIPN experienced several emotional challenges such as feelings of isolation, frustration, depression, and loss of purpose, due to the inability to perform daily activities [17, 18]. CIPN was described as a background noise and as a constant reminder of the cancer disease [17]. Even as a background noise, CIPN was distracting and demanding, affecting the emotional and physical well-being, and interfering valued activities, relationship with friends, family, and work, and the ability to participate in daily activities [18].

The above findings suggest that CIPN has serious consequences on the physical, psychological, and social aspects of haematological cancer survivors’ everyday lives. However, these studies [17, 18] did not only study the perspectives of haematological cancer survivors but also survivors of various cancer types. Furthermore, it is difficult to distinguish whether there is a difference between the different groups and their experience with CIPN in everyday life. The current research does not make sure to inform healthcare professionals sufficiently of how it is to live with CIPN. This study aims to explore and gain an understanding of haematological cancer survivors’ experience with CIPN in their everyday life.

## Methods and design

This qualitative study employed thematic analysis of semi-structured interviews with haematological cancer survivors to explore their experiences of living with CIPN. A phenomenological-hermeneutic approach was used to obtain in-depth descriptions of CIPN and interpret the meaning through prominent themes. The phenomenological approach is based on the philosopher E. Husserl’s phenomenological philosophy of pure descriptions of a given phenomenon [19] and guided the data collection. The hermeneutic approach is based on the philosopher Hans-Georg Gadamer’s hermeneutic philosophy, where understanding is an interpretation [20], processed in a thematic analysis [21, 22]. The study is reported according to consolidated criteria for reporting qualitative research (COREQ) [23] as shown in the supplementary file (Supplementary file 1).

### Setting and participants

The participants were recruited from a Haematological Outpatient Clinical at a public Danish University Hospital and the Danish Patient Association of Late Effects. Purposeful sample strategy [24] was used to recruit participants with experiences relevant for aim of the study, and physicians were used as gatekeepers in the outpatient clinic. Convenience sampling was also applied [24], as participants also were recruited from the patient association by a posting on their social media. The inclusion criteria were adults ≥ 18 years, who had received neurotoxic chemotherapy for a haematological cancer disease (i.e. lymphoma, multiple myeloma, or leukaemia). They had been assessed by a physician or themselves having tingling, burning, numbness, stinging, or painful sensations in the lower and/or upper extremities — symptoms that were not present prior receiving cancer treatment. Furthermore, they had to be able to read and speak Danish. The exclusion criteria were participants with pre-existing neuropathy, e.g. related to diabetes or neurological diseases unrelated to chemotherapy, and participants with cognitive impairment.

### Data collection

The semi-structed interviews were conducted between March and July 2023, at a time and location (home, hospital, online, or by phone) chosen by the participants, with the purpose of creating a safe atmosphere allowing the participants to express themselves freely [25]. A semi-structed interview guide based on open-ended questions was used to capture the participants’ experiences of everyday life with CIPN, see Table 1. An open and naïve approach was used to (further) explore the participants’ responses, paying attention to their non-verbal communication and asking probing questions, e.g. by using mirroring and paraphrasing. The interviews were conducted by the first author (nurse with a master’s degree and with experienced haematological nursing). The first author had no former relationships with the participants prior to study commencement, and no one else was present during the interview besides the participant and the interviewer. The interviews were audio recorded and lasted from 31 to 63 min (average 43 min).

**Table 1:** Interview guide

1. In your own words, tell me about your experiences with neuropathy after completion of chemotherapy?
2. How does the neuropathy manifest itself? (numbness, tingling, burning, stinging or painful sensations).
3. Which words would you use to describe your neuropathy?
4. How would you describe a typical day with neuropathy?
5. In what way does neuropathy affect your ability to perform everyday activities? Can you give me some examples? (E.g., when you move around, leisure activities, work, and other things).
6. Tell me in your own words how neuropathy has affected the way you perceive yourself? Please describe how you experience this.
7. Tell me in your own words how neuropathy has affected your social relations (E.g., in relation to your spouse, children, family, friends and colleagues).

### Data analysis

The interviews were transcribed verbatim, and data were analysed using thematic analysis in a six-phase data-driven process inspired by Braun and Clarke [21, 22]. The thematic analysis is a method for identifying, analysing, and reporting themes that turn out to be important to the study aim and is closely related to the data [21].

In phase one, the first author listened to the interviews and read the transcripts several times to become familiar with the wide and depth of the data. In phase two, initial codes were generated manually by reading the transcripts line by line. In this phase, quotes relevant for the study aim were assigned one or more codes and a coding tree was produced, see Table 2. In phase three, three of the authors (MLR, LJ, and HE) searched for patterns across all coded data and formulated tentative themes. In phase four, the tentative themes were discussed in an iterative process between the previous phases to clarify and present the essence of each theme. This process led to four themes. In phases five and six, the themes were refined. Themes were defined and named, and quotes assigned to each theme were interpreted and transformed into descriptions supported by selected quotes.

**Table 2:** Coding tree of the qualitative interviews

Initial codes	Preliminary themes	Themes
Difficult to describe Analogies / metaphors A strange and unpleasant sensation on/off The expression of neuropathy Numb but painful and hypersensitive Constantly present An unclear experience	The peculiar and ambiguous expression of CIPN An experience which is difficult to describe Body discrepancy A constant reminder	A diffuse and contradictory sensation which is impossible to ignore in everyday life
Reminder of cancer CIPN as a consequence Everyday challenges Work ability Lack of sleep Social relations Family and friends Change of identity Being unwell Psychological impact Quality of life Gratitude for being alive A price for being alive	Being unwell, despite being cured A price for being alive Changes in relationship (physical, psychological, and social) A changed self-understanding	Not feeling well, even though I’m cured
Learning to live with it Uncertainty Concern Interventions Coping Accept	Living with CIPN To accept the situation Handling of concerns and uncertainty Adaption to CIPN	Living with CIPN, despite limitations
Focus on late effects A call for help Lack of help Communication with healthcare professionals CIPN as a lower priority	A hidden disorder Lack of understanding and recognition	An invisible companion, that everybody knows about

### Ethical consideration

The study complies with the ethical guidelines for research of The Nordic Nurses Federation [26] and the Helsinki Declaration [27]. Approval from the National Ethics Committee was not required, as interview studies not including human biological material do not have to be reported according to Danish legislation [28]. The participants were informed orally and in writing about the study and signed an informed consent before the interview. The participants were informed that participation was voluntary, and they were guaranteed anonymity and the right to withdraw from the study. Data was stored securely and was only accessible to the researchers.

## Results

12 participants in total contacted or were referred to the first author (MLR) and all consented to participate in the study. They represented diversity in age, gender, social status, working status, haematological cancer, and time since treatment cessation, see Table 3.

**Table 3:** Characteristics of the participants

ID	Age	Gender	Social status	Working status	Cancer type	Time between interview and treatment cessation
1	49	Female	Married	Full-time employment	Acute myeloid leukaemia	19 years and 6 months
2	64	Male	Married	Disability pensioner (due to CIPN)	Multiple myeloma	4 years
3	46	Female	Married	Job capacity assessment (due to CIPN)	Lymphoma	1 year and 1 month
4	67	Female	Married	Working part time	Multiple myeloma	2 years and 5 months
5	51	Female	Cohabiting	Flexible employment	Lymphoma	4 years and 5 months
6	73	Female	Married	Retired	Multiple myeloma	3 years and 11 months
7	67	Female	Married	Retried	Acute promyelocytic leukaemia	4 years and 7 months
8	75	Female	Married	Retried	Multiple myeloma	10 years and 6 months
9	55	Female	Married	Sick leave	Lymphoma	8 months
10	68	Female	Married	Retried	Multiple myeloma	2 years
11	74	Male	Married	Retried	Multiple myeloma	10 years
12	85	Male	Married	Retried	Lymphoma	4 years and 11 months

The interviews were conducted at the participants’ private homes (*n* = 4), or at the hospital in a suitable room (*n* = 3), online via Zoom (*n* = 3), and on the phone (*n* = 2). Despite the variation in the participants including the time since treatment sessions, they all experienced that everyday life had been changed by CIPN, which is described in the four themes: (1) *A diffuse and contradictory sensation which is impossible to ignore in everyday life*, (2) *Not feeling well, even though I’m cured*, (3) *Living with CIPN, despite limitations*, and (4) *An invisible companion that everybody knows about*. Although the four themes are presented as separate themes, they must be seen as a unified entity in response to the aim of the study.

### A diffuse and contradictory sensation which is impossible to ignore in everyday life

This theme describes and gives an understanding of how the participants perceived CIPN as a diffuse and contradictory sensation which was impossible to ignore in everyday life.

The participants had difficulty putting CIPN into words. Some participants struggled to describe the experience of CIPN and used words like “strange” and “weird”, and others could not express what CIPN feels like. One participant said: “*It’s that diffuse thing you can’t describe*.” (ID 1). Other used words such as “*frozen fingers and toes*”, “*swollen and sunburnt hands*”, and “*numbness*”, “*burning*”, “*prickling*”, and “*tingling*” sensations in hands and feet to describe CIPN. Some participants even described CIPN as if the skin was penetrated by knives, glass, gravel, stones, or needles. One participant said: “*It’s prickling and tingling, as if thousands of needles are stuck in me*.” (ID 8). The participants used metaphors to be able to explain their experiences of CIPN that they otherwise had difficulty expressing.

Moreover, the participants generally experienced CIPN in their hands and feet and highlighted that they felt hypersensitive and numb at the same time. “*It’s like they’re [the feet] hypersensitive, and at the same time I’ve lost some of the feeling in them. How does it sound? Sounds silly, right?*” (ID 6). Experiencing contradictory sensations of CIPN simultaneously indicated the diffuse sensation of CIPN. In addition, the participants highlighted that although their hands and feet looked normal, they felt enlarged, swollen, and tense. One expressed the experience as follows: “*Sometimes I must look down at my feet. It feels like my feet are swollen. So tense, you know. But they are not. They are completely normal. But it’s feeling*.” (ID 6). The participants described a contradiction which illustrated how they experienced their bodies different from the way it physically appeared to them. This indicates that the experience of CIPN contains a contradiction between the physical body and the perception of the body, which seems difficult for the participants to relate to in their everyday life.

Furthermore, the participants described that they were conscious of CIPN all the time — both day and night. One said: “*They [the sensory disturbances] are there more or less all the time.” (ID 7). CIPN was present even when they were resting: “I feel them [the sensory disturbances] from the moment I open my eyes in the morning. I’m never in doubt that I have legs or hands. Because they are there all the time. And at night. I feel it 24 hours a day*.” (ID 8). CIPN was also present when they were active. One participant expressed his experience of walking: “*It is like walking on broken glass. It simply hurts my feet so much that I cannot do it*.” (ID 2). This indicates that the participants’ awareness was constantly focused on these sensations of CIPN that was always present and impossible to ignore in everyday life. In this way, CIPN affected several aspects of everyday life for haematological cancer survivors — as a diffuse and contradictory sensation which was all pervading.

### Not feeling well, even though I’m cured

This theme illustrates the participants’ experience of being cured of a haematological cancer disease, and at the time same feeling unwell due to CIPN.

Not feeling well, despite being cured, was highlighted by the participants in different ways. One said: “*I was not aware that there could be this [CIPN] afterwards. The focus is to get well, but I’m surprised that you get ill from the treatment you have received*.” (ID 3). Having CIPN as a consequence of the cancer treatment was unexpected to some of the participants and CIPN made them feel unwell. Others described CIPN as the price for being cured: “*It’s a price. It’s a price you pay to be allowed to be here*.” (ID 7). Although the participants were thankful for being alive, they still felt that CIPN impacted their life. One participant said: “*It [CIPN] should not overshadow the rest of my life. It’s only good to have it. Life*.” (ID 4). This indicated that CIPN was a downside of being cured of haematological cancer. For others, CIPN had constantly reminded them that they had suffered from haematological cancer. One participant said: “*You’re reminded of it [that I had cancer] every single day, and you just hope that it [CIPN] will get better*.” (ID 3). This indicated that CIPN became an eternal reminder of their haematological cancer because CIPN was a consequence of the cancer treatment.

The participants described different activities of everyday life that were no longer possible or were negatively impacted due to CIPN. They talked about being restricted, which made them feel like they were still ill. “*I am restricted in terms of movement. There are many things, which just can’t be done anymore. It makes me feel like I’m still ill. It’s terrible that you can’t do the things that you have done before*.” (ID 4). For several participants, CIPN prevented them from doing basic physical things like walking, due to problems with balance, fall, or cramps. One formulated it as follows: “*Something like going for long walks. I can’t do it anymore. I get so many cramps that I can’t move at all. It is one of the things I’m sad about, because I liked going for walks*.” (ID 8). Others described reduced fine motor skills affecting their ability to dress and do household duties, like cooking, doing the laundry, and cleaning. In addition, to affecting the physical dimension, CIPN also affected the psychological and social dimensions of everyday life. Several described that their everyday life was characterised by lack of sleep due to cramps, and others could no longer participate in their leisure activities. One participant who used to enjoy her work as a chef was no longer able to work due to CIPN in her hands. “*It actually means a lot to me, that I can’t do this anymore [work]*.” (ID 3). The changes in everyday life affected the participants’ physical, psychological, and social well-being. Having CIPN forces the participants to see themselves in a new way. Some are preoccupied with the things they can no longer do; others find alternative ways of living in everyday life. These contrasts illustrate an everyday life with bodily limitations, which clarify the participants’ new ways of being in the world as being unwell, despite being cured.

### Living with CIPN, despite limitations

This theme demonstrates how the participants in different ways are living with CIPN in their everyday life.

Several participants used a variation of the phrase: “*I’ve learned to live with it*” to illustrate how they cope with CIPN in everyday lives. Some expressed that they had to adapt their everyday life, where pain was constantly present. “*I have learned to live with the pain being there all the time. Now four years have passed and that’s how it is. You learn to live with it*.” (ID 2). This indicated that some participants accepted pain as a part of their everyday life. Others tried to live their everyday life in a way where they were not limited by CIPN. “*I do what I can possibly do. I will not be limited*.” (ID 8). The participants used different approaches to handle CIPN and showed how they did things differently to avoid that CIPN limited them in their everyday life. They talked about ignoring, controlling, or minimising the negative impact of CIPN. One woman described how she tried to control her everyday life: “*If it doesn’t work out in one way, it might work out in another way. Why should we make restrictions if we can do it another way*.” (ID 7). This strategy shows that some participants adjusted or modified their everyday life to minimise CIPN’s negative impact. Others planed their everyday life around CIPN, as they were conscious about how different activities increased or minimised the symptoms of CIPN. For example, they planned periods of rest as a perquisite for being able to participate in physical and social activities. “*I try to plan my everyday life when I am active. Then I am thinking in the long term. How can I relieve [my body] to complete this [activities]?*” (ID 9). The participants tried to do the things they valued in everyday life by planning and knowing that it had consequences. This balance between rest and activity required adaption, stubbornness, and fragility.

Although several participants described how they endured the pain of CIPN, they also expressed worries about CIPN. One participant reflected: “*Will it get even worse?*” (ID 10). The participants were worried whether the symptoms of CIPN would get worse, change, or never disappear. Some told that knowing about CIPN helped them minimise their worries. One participant said: “*I know what it is. If I know what it is, it’s not like I’m going to worry about it. I know where it comes from and why*.” (ID 1). This indicates that worries about the long-term impact on CIPN also affected their everyday life and that some could manage these worries but also endure CIPN by knowing about CIPN.

### An invisible companion that everybody knows about

This theme shows that CIPN is like an invisible companion that everybody knows about, but not always understand or address, which made the participants feel alone with CIPN.

The participants did not experience that others understood how CIPN impacted their everyday life. One participant said: “*Sometimes I think that if only there were others who could understand how … those in my surroundings. If only they had to try one day [to have CIPN]. Just one day, so they would know how it’s like*.” (ID 7). Being the only one who fully understand how it’s like to have CIPN seems to make the participants feel alone with CIPN. The fact that others cannot fully understand what it is like to have CIPN seemed to reinforce the participants’ feelings of being alone with CIPN and the consequences it has for their everyday life.

Several participants expressed that they had received sufficient help from healthcare professionals to manage the side effects during treatment with chemotherapy but felt that they did not receive help to manage CIPN after their cancer course. “*I find it difficult to accept that having received cancer treatment, you get some other problems and then … well, it’s just the way it is. I can’t accept that. Could you do something, and then say that you have done what you could. But no one has done anything. But apparently that’s how it is. I don’t’ know what can be done and by whom*.” (ID 2). Standing alone with CIPN after cancer treatment seemed like an integral part of the participants’ everyday life and illustrated how they had to navigate on their own in a new everyday life with CIPN. During the follow-up period, participants described that CIPN was perceived as a lower priority compared to the haematological cancer disease and other symptoms in the outpatient unit. One participant said: “*It [CIPN] was not prioritised*.” (ID 5). Despite reporting their CIPN symptoms to the healthcare professionals, the participants described that they received little consulting or help to manage CIPN and rarely did the healthcare professionals assess how CIPN affected their daily life. “*It has not been taken care of. At all. Therefore, I hope that there will be others who will receive help from day one*.” (ID 5). Several participants experienced being alone with CIPN, and they indicated that the healthcare professionals should address CIPN to help them not feeling alone with the symptoms and even help them how to deal with CIPN in their everyday life. This indicates that the participants expect the healthcare professionals to address CIPN. Although the participants tried to make CIPN visible to their relatives and the healthcare professionals, it was still invisible. It was perceived as an invisible companion that only they understood, and that there was not enough focus on CIPN in the healthcare system following a cancer course.

## Discussion

The study aimed to explore haematological cancer survivors’ experience of CIPN in everyday life after a haematological cancer disease. The findings make an important contribution to the knowledge about everyday life with CIPN among haematological cancer survivors and highlight the impact of CIPN in the transition to an ordinary everyday life following a cancer treatment.

In this study, it became clear that survivors with haematological cancer found it difficult to express the sensation of CIPN, but using metaphors facilitated describing their experiences. These findings align with earlier studies, showing that cancer survivors have difficulty putting CIPN into words [17, 29–31]. However, using metaphors made it possible to capture CIPN symptoms [17, 30, 32, 33]. When the healthcare professionals explore the symptoms of CIPN, it is important that they are aware of haematological cancer survivors’ use of metaphors as that could explain how CIPN is experienced.

Moreover, the findings indicated that CIPN has an impact on everyday life activities among haematological cancer survivors which correspond with findings among other cancer survivors with CIPN [16, 29–33]. A paradoxical feature of cancer survivors is that they do not necessarily see themselves as fully recovered, despite being cured [30, 34, 35], which was also the conclusion in our study. Thus, the present findings show that the bodily changes of CIPN are not only affecting the living circumstances, but also haematological cancer survivors’ well-being. Their self-understanding has been changed, and the body is no longer mute, but is in in an everyday life, which make them feel unwell. Our study revealed that despite being cured of haematological cancer, CIPN interfered with both the physical, psychological, and social aspects of everyday life. CIPN was perceived as a price for being alive, but the freedom to live their lives as usual was affected. This indicates that haematological cancer survivors recognise their new self-understanding as being ill following chemotherapy, which can be interpreted as illness because it illustrates their subjective experience of still being unwell, despite being cured. According to Kleinman, “*illness*” refers to the subjective experience of symptoms and suffering [36]. It is therefore essential that the healthcare professionals are aware of haematological cancer survivors’ experience of illness and not only focus on the symptoms of CIPN, which are expressed in the concept of “*disease*” as a biophysiological abnormality of the body [36].

The findings also showed that haematological cancer survivors experienced CIPN as a downside of being cured. This may indicate that CIPN challenges the transition to an ordinary everyday life following haematological cancer treatment, because CIPN may have a major impact on several aspects of everyday life. Thus, the healthcare professionals have an important role in addressing CIPN as it affects their well-being and quality of life. Research shows that CIPN has a negative impact on quality of life for haematological cancer survivors because CIPN results in severe debilitation of physical, psychical, and social character [9–12, 37, 38]. This emphasises the importance of CIPN being addressed in the follow-up period to support haematological cancer survivors’ transition to an ordinary everyday life.

In general, CIPN appeared as a living condition that led to limitations, adaptations, and worries. The findings showed that haematological cancer survivors managed CIPN differently. This can be seen as different coping strategies. Coping is described as constantly changing cognitive and behavioural efforts to manage specific external and/or internal demands that are appraised as taxing or exceeding the resources of the person [39]. Our findings showed that haematological cancer survivors adapted to everyday activities using cognitive and behavioural processes such as ignoring, controlling, or minimising symptoms to keep CIPN at bay. These strategies described how haematological cancer survivors cope with CIPN in everyday life. Depending on symptom intensity, haematological cancer survivors could use more than one of these strategies to keep CIPN more into the background. In many ways, our findings are similar to the findings that have been found among cancer survivors in general [17, 33, 40]. This suggests that the experience of CIPN in everyday life does not depend on the specific cancer diagnosis and must be seen as a general living condition after a cancer disease. When healthcare professionals have to support cancer patients with CIPN, they should offers support for each individual cancer survivor and not be preoccupied of the specific cancer diagnosis.

The interviews revealed that haematological cancer survivors often feel alone with CIPN, i.e. there is no follow up when mentioning their CIPN symptoms to healthcare professionals. Previous studies report that cancer survivors’ experience that healthcare professionals primally focus on the cancer treatment and survival; they are too busy; they have no idea that CIPN is common; and they only notice CIPN when the patients cannot cope with the symptoms anymore [16, 32]. Haematological cancer survivors do not feel seen and heard in relation to CIPN and the impact on their everyday life. For haematological cancer survivors, it seems important to be met when struggling with CIPN, even if current treatment cannot minimise the symptoms. Being alone with CIPN is challenging. The findings of loneliness, while not unexpected, adds something unique to haematological cancer survivors and is an important message for healthcare professionals who are working with haematological cancer survivors in clinical practice. However, to fully understand the collaboration between healthcare professionals and haematological cancer survivors regarding CIPN, it is important to explore the barriers and facilitators addressing CIPN from the perspective of healthcare professionals and haematological cancer survivors in future research.

### Strengths and limitations

The variation in the participants’ demographic data is a strength as this included a deeper and more nuanced understanding of haematological cancer survivors’ experience of living with CIPN in everyday life. Since the study contains a focused aim and all participants (*n* = 12) had relevant experience in relation to the aim of the study, the number of participants is considered satisfactory because the participants had high information power regarding the aim of the study [41]. The fact that the participants had different haematological diagnoses may be considered both a limitation and a strength of the present study. The findings may have been more specific if the participants had shared the same diagnosis. On the other hand, it was also a strength that there was a diversity in the participants’ haematological diagnosis which was reflected in the clinical practice where haematological cancer survivors were often seen in the same outpatient clinics.

Mirroring and paraphrasing were important tools in a phenomenological-hermeneutic inspired interviews [42]. Therefore, it can be considered a limitation that five interviews were conducted online or via telephone, as it was not possible for the interviewer to mirror or react to the informant’s non-verbal communication. On the other hand, it was important to conduct the interviews according to the informant’s decision to create a safe atmosphere, which allowed them to express themselves freely. Another strength of the study was that the steps of the analysis were presented explicitly to achieve transparency, and that the analysis was continuously discussed with the co-authors in order to increase validity of the findings [43]. Overall, the study is considered credible, and it is up to the reader to assess whether the findings are transferable to other populations and contexts [43].

## Conclusion

This study gives voice to a serious late effect following haematological cancer treatment and contributes to a better understanding of everyday life with CIPN among haematological cancer survivors. It can be concluded that survival from haematological cancer not always equals well-being, as experiencing CIPN has extensive consequences on everyday life and thus appears as a living condition. CIPN is a diffuse and contradictory symptom that impacts haematological cancer survivors’ everyday life. However, haematological cancer survivors adapt to everyday life even though CIPN causes disturbances in the physical function, daily activities, social relationships, psychological aspects, and work ability. CIPN appears as an invisible symptom and a traveling companion and is dominated by limitations, adaptions, and worries. This often leads to a feeling of lack of understanding causing the experience of being alone with this influential symptom.

### Clinical implications

The National Cancer Research Institute has identified the consequences of nerve damage caused by cancer treatment as one of its top research priorities [44]. While neurotoxic chemotherapy enters as an integral part of the treatment of haematological cancer, and as more individuals survive, the incidence of CIPN among haematological cancer survivors will increase during the coming years. The importance of healthcare professionals’ knowledge and understanding of everyday life with CIPN should be recognised in order to provide adequate care and treatment of haematological cancer survivors’ needs. In clinical practice, it is important to consider strategies that enable haematological cancer survivors to identify the existence of CIPN-symptoms early in the treatment and after treatment, to report their symptoms to the healthcare professionals, and to support them in managing their symptoms. However, it is also recommended that during the follow-up period, the healthcare professionals should identify and address CIPN as a living condition of everyday life. There is a need for increased attention to articulate CIPN in everyday life. Further, healthcare professionals can help haematological cancer survivors not feeling alone by exploring their experience with CIPN in everyday life and its impact of their well-being, e.g. by focusing on haematological cancer survivors’ experience of illness despite being cured. Healthcare professionals should recognise that CIPN is not only a physical symptom, but also affects the psychical and social well-being and quality of life. In addition, it is important to assess the individual cancer survivors’ information needs and tailor the communication, advice, and guidance, as they manage CIPN differently in everyday life.

## Electronic supplementary material

Below is the link to the electronic supplementary material.

Supplementary Material 1

## Data Availability

The de-identified qualitative dataset used in the analysis are available from the first author (MLR) on reasonable request.
